# Characterizing Acoustic Behavior of Silicon Microchannels Separated by a Porous Wall

**DOI:** 10.3390/mi15070868

**Published:** 2024-06-30

**Authors:** Mehrnaz Hashemiesfahan, Jo Wim Christiaens, Antonio Maisto, Pierre Gelin, Han Gardeniers, Wim De Malsche

**Affiliations:** 1 µFlow Group, Department of Chemical Engineering, Vrije Universiteit Brussel, 1050 Brussels, Belgium; jo.wim.christiaens@vub.be (J.W.C.); antonio.maisto@vub.be (A.M.); pierre.gelin@vub.be (P.G.); 2 Mesoscale Chemical Systems Group, MESA+ Institute for Nanotechnology, Faculty of Science and Technology, University of Twente, 7500 AE Enschede, The Netherlands; j.g.e.gardeniers@utwente.nl

**Keywords:** acoustofluidics, porous wall, acoustic streaming, micromembrane

## Abstract

Lateral flow membrane microdevices are widely used for chromatographic separation processes and diagnostics. The separation performance of microfluidic lateral membrane devices is determined by mass transfer limitations in the membrane, and in the liquid phase, mass transfer resistance is dependent on the channel dimensions and transport properties of the species separated by the membrane. We present a novel approach based on an active bulk acoustic wave (BAW) mixing method to enhance lateral transport in micromachined silicon devices. BAWs have been previously applied in channels for mixing and trapping cells and particles in single channels, but this is, to the best of our knowledge, the first instance of their application in membrane devices. Our findings demonstrate that optimal resonance is achieved with minimal influence of the pore configuration on the average lateral flow. This has practical implications for the design of microfluidic devices, as the channels connected through porous walls under the acoustic streaming act as 760 µm-wide channels rather than two 375 µm-wide channels in the context of matching the standing pressure wave criteria of the piezoelectric transducer. However, the roughness of the microchannel walls does seem to play a significant role in mixing. A roughened (black silicon) wall results in a threefold increase in average streaming flow in BAW mode, suggesting potential avenues for further optimization.

## 1. Introduction

Microfluidics, a pivotal tool in substance separation science, has evolved significantly since its inception. Initially, it was used to flow microvolume samples in the channel and separate chemical compounds and molecules by chromatography [1]. The first microfluidic devices were silicon channels closed by glass, and later, other materials were introduced for further exploration [2]. The applications of separation technologies in microfluidics are vast, spanning from separating bioparticles such as DNA, proteins, and exosomes in biomedical engineering [3] to water purification and industrial processes [4]. A substantial portion of separation studies focuses on high-throughput separation, which has potential applications in drug development, diagnostics, biosensing, and genomics [5]. This broad spectrum of applications underscores the importance and versatility of microfluidics in substance separation science.

In microfluidics, separation methods can be divided into active and passive methods. Active methods usually rely on external forces such as magnetic, electrical, optical, and acoustic forces to control and improve the separation performance [1].

In contrast, passive methods mainly depend on the hydrodynamic characteristics of the designated microfluidic chip [1]. An example of this technique is ultrafiltration, which uses designs in microfluidics to separate the chemicals based on their size, weight, and charge. Ultrafiltration and microfiltration both use various kinds of membranes for filtration [2]. The microfluidic devices for these purposes usually have a part called the microfluidic membrane mimic (MMM) that mimics the structure and function of a membrane by having pillars, curvatures, or other structures resembling tiny pores [3]. Of the most used materials for fabricating porous membranes, polydimethylsiloxane (PDMS), cellulose-based materials, polyethylene (PE), and poly(vinylidene fluoride) (PVDF) can be mentioned [4]. Of the less popular materials for fabricating porous membranes, silicon has its own unique benefits, such as precise control of pore size with micromachining methods, unique mechanical properties, and chemical compatibility (especially if it is passivated with, e.g., a silicon oxide layer) [5].

Both active and passive separation methods have advantages and disadvantages, but combining both the active and passive strategies in a system can give promising results in the cases of efficiency and selectivity [6]. This hybrid system takes advantage of both approaches: for instance, in continuous cell sorting development, if a hybrid system is used, it is possible to separate particles at lower flow rates, while in a passive system, a higher flow rate is needed [7]. Since using a hybrid system has such an impact on efficiency, a hybrid system of various kinds of membranes in a microfluidic chip combined with an external force to induce faster separation has drawn attention. For instance, by combining a nonporous membrane and electrophoresis in a microfluidic filtration system, extracellular vesicles can be separated from whole blood in a higher throughput manner [8]. This study falls under the category of a combination of both active and passive separation methods, specifically exploring the use of acoustic forces to enhance lateral transport in microchannels.

An essential requirement to enable fast separation is that the contact time between species in the channel and the separation zone (the membrane) is short [9]. As is mostly the case in microfluidics, the flow regime of the devices is laminar, and in a laminar flow regime, fluid layers move parallel to one another with minimal mixing, except through diffusion [10]. Reducing the channel size reduces the lateral transport time, but to achieve industrially relevant flow rates and minimize clogging, especially when working with particles, channel dimensions in the range of 100 s of µm are mandatory.

Acoustofluidic devices refer to the use of sound waves to manipulate micron-sized objects and environments within the designated channel. These waves have wavelengths larger than the sizes of bioparticles, allowing for high-precision manipulation of particles or cells thanks to scattering. These waves can be generated through mechanical vibrations, such as those induced by the piezoelectric effect, and offer advantages such as straightforward device designs, biocompatibility, fast liquid flow distribution, and compactness, making them ideal for diagnostics, therapeutics, and industrial applications [11]. This multidisciplinary field that integrates acoustic forces with microfluidic devices and fluid dynamics is referred to as acoustofluidics [12].

Bulk acoustic waves (BAWs) and surface acoustic waves (SAWs) are two types of acoustic waves used in acoustofluidics. BAWs are mechanical waves that propagate inside a cavity, while SAWs are acoustic waves that travel along the surface of an elastic material. BAWs are categorized into longitudinal and transverse waves, while SAWs are typically shear waves coupled with longitudinal ones [13]. Standing BAWs are generated by a piezoelectric transducer that, with the appropriate conditions, excites standing waves in microfluidic cavities at geometrically dependent resonant frequencies which can provide a gentle and non-invasive method for particle manipulation and separation, improving the filtration process’s efficiency and throughput [14]. The use of inter-digited transducers commonly produces SAWs. SAWs propagate along the surface of the material, while BAWs propagate inside the bulk of the material [15]. 

Using BAWs has some advantages over SAWs: SAWs travel along the surface of the material, leading to a limited penetration depth into the substrate. This limitation will affect the interaction with particles or cells that are not in close proximity to the surface, resulting in a reduction in the effectiveness of manipulation and separation. The wave propagation in anisotropic materials like silicon, which is relevant to BAWs, is more complex than in isotropic materials such as PDMS, which is more elastic than silicon and widely used for applying SAWs inside a microfluidic channel. This complexity can pose challenges in the design optimization and material choice for SAW-based microdevices [16]. In addition to all the points mentioned earlier, BAWs are less sensitive to surface roughness compared to SAWs, which require a smooth surface for optimal performance. Such considerations make BAWs more suitable for applications involving materials with rough or textured surfaces [15]. Standing bulk acoustic waves in a rectangular channel creates two effects: the liquid streaming generates a drag force on the particles and the scattering of acoustic pressure waves in the liquid results in a radiation force along the width of the channel. The magnitude of each force inside the channel is dependent on various factors, including the resonance frequency and particle size [17].

In the present study, standing BAWs are induced in a silicon microfluidic channel divided by a fabricated membrane formed by an array of rectangular pillars with 2.5 µm spacing, performing the function of a thin wall with pores. The effect of the applied potential at the PZT actuator on vortex flow formation and average velocity magnitude has been studied. Part of this study focuses on enhancing the vortex flow velocity by increasing the channel roughness. The last part is dedicated to the effect of pore size on the magnitude of acoustic radiation force inside the channel, providing experimental results as well as a simulated model.

## 2. Materials and Methods

### 2.1. Acoustofluidic Setup

**Figure 1:**
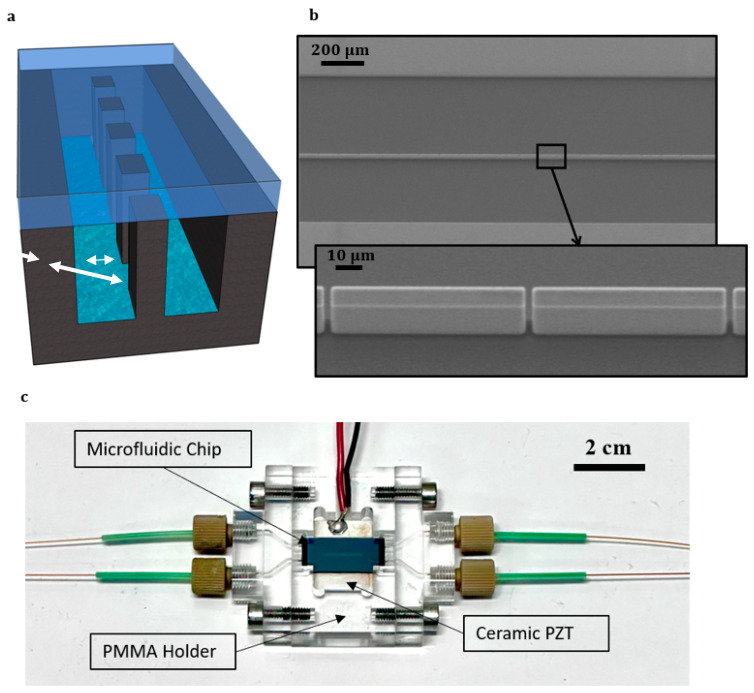
The microfluidic chip tested with a porous wall: (**a**) the schematic of the microchip design and (**b**) a tilted SEM photo from the top of the microfluidic chip show the rectangular pillars that are formed as a membrane between two channels (W: 375 μm, D: 50 μm) with the porous wall (thickness: 10 μm, pore size: 2.5 μm); (**c**) photograph of the complete acoustofluidic setup.

The microfluidic chip consisted of a microchannel (10 mm × 760 µm × 50 µm) in silicon with a silicon membrane in the middle. The silicon membranes were made of rectangular pillars in two sizes, (20 µm × 10 µm × 50 µm) and (80 µm × 10 µm × 50 µm). The space between pillars had two varieties, 2.5 µm and 5 µm. The design of the device was achieved through a single-layer mask transferred to a positive photoresist through lithography and etched with a Bosch-type dry etching process. The silicon chip was sealed with a MEMpax^®^ borosilicate glass wafer by anodic wafer bonding and located with the silicon side on top of a ceramic piezo element (20 mm × 15 mm × 1 mm, APC International Ltd., Mill Hall, PA, USA) with a thin layer of glycerol in between as a coupling agent. The piezo element generated a 2 MHz resonance frequency by a frequency generator (AFG1062, Tektronix UK Ltd., Berkshire, UK), and the applied voltage was amplified 340 times by an RF power amplifier (210 L, Acquitek S.A.S, Prana, France) with a power output of 10 Watt. The setup parts were connected with the help of multiple PMMA holder pieces. Figure 1 depicts the complete overview of the microfluidic chip and the assembly of the acoustofluidic setup.

### 2.2. Particle Focusing Experiments

For particle experiments, fluorescent monodispersed polystyrene particles 5 µm in size (Microparticles GmbH, Taufkirchen, Germany), diluted to 0.001 *w*/*v*% in water, were pumped into the channel. Microscope movies were recorded while applying acoustic force to the chip. A set of movies was made at a 2 MHz resonance frequency and an applied voltage of 34–136 V.

### 2.3. Determination of Average Velocity Magnitude of the Acoustic Streaming

Fluorescent monodispersed polystyrene particles 1 µm in size (Microparticles GmbH, Germany), diluted to 0.005 w/v% in water, were pumped into the channel, and movies were recorded while applying acoustic force to the chip. A set of movies was made at a 2 MHz resonance frequency and an applied voltage of 13–41 V. The velocity of particles as a function of their position in the channel was studied and processed with a customized MATLAB code. The code calculates the velocities by dividing the differences between consecutive positions by the time step.

### 2.4. Evaluation of Mixing Effects Related to Acoustics Streaming at 1 MHz and 2 MHz

A 20 KDa Fluorescein isothiocyanate–dextran (Sigma Aldrich, Burlington, MA, USA) in a concentration of 1 mg/mL was used to track diffusion. One of the channels was loaded with Fluorescein, while the other was loaded with pure water. As soon as the liquid interface was stable at the wall, the flow was stopped, and the diffusion was evaluated. The intensity of the fluorescence in the picture is proportional to the concentration [18]. Then, using the same methodology, mixing was evaluated by actuating 1 MHz and 2 MHz ls at 34 Vpp. Such values were chosen to avoid longitudinal resonance and have a relevant effect, generating a secondary flow at a higher actuating voltage. The variation of the intensity of fluorescence was evaluated through ImageJ (version: 1.53e; Java 1.8.0_172) over the entire width of the channel.

## 3. Results and Discussion

**Figure 2:**
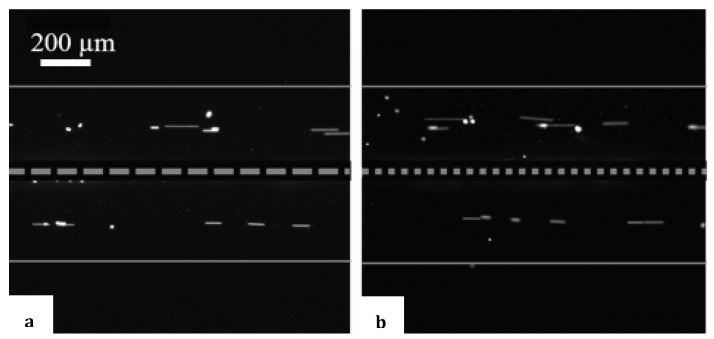
Focusing of 5 µm polystyrene particles in the middle of each channel under the influence of acoustic radiation force: (**a**) larger pillars with 12 pores/mm (Supplementary Video S1), (**b**) small pillars with 80 pores/mm (Supplementary Video S2). (The grey squares are intended solely to give a better understanding of the membrane position; they are not to scale).

One of the first experiments performed with the assembled acoustofluidic setup was to validate that at the resonance frequency that was being used, a standing bulk acoustic wave was effectively generated inside the microfluidic chip. This way, the amplitude of the acoustic field could be maximized, resulting in higher acoustic effects on the particles. This allowed for more efficient particle manipulation [19]. The standing wave created a region of intense acoustic pressure in the liquid, which could be used to focus ‘hard’ particles in a specific location (pressure nodes) in the channel [20]. A focusing effect indicates whether the proper resonance dimension and frequency combination has been implemented. Figure 2 displays the focus line of 5 µm polystyrene particles inside the channel. Although there is a porous membrane in the middle of the 760 µm channel, the results indicate that in the case of focusing the particles, the permeable membrane acts as a solid wall in the middle, physically dividing the channel into two channels of 375 µm.

The particles focus in the middle of each channel, forming a straight line at 2 MHz, assuring the formation of a standing wave in the channel. In water, sound velocity equals 1.5 × 10^3^ m/s at a frequency of 2 MHz, which corresponds to a wavelength of 760 µm. Based on this indication, at a 2 MHz resonance frequency, 5 µm polystyrene particles only focus in the middle of 375 µm channel under two circumstances: (1) if the membrane would be considered to separate the channels not only physically but also acoustically (one pressure node at 2 MHz in each channel of 375 µm wide) or (2) if the membrane were considered to be acoustically transparent despite dividing the device physically (two pressure nodes at 2 MHz in a 760 µm-wide channel). In either circumstance, the number of nodes at 2 MHz will be the same if the membrane is transparent to acoustic waves or not, as the first harmonics for a 760 µm channel will correspond to the fundamental harmonic of the 375 µm channels.

In order to discover whether the membrane used in the microdevice in this research was acoustically transparent, focusing experiments with 5 µm polystyrene particles were repeated, but this time, instead of a 2 MHz resonance frequency, 1 MHz was used. In such a way, only one node would be produced in the 760 µm channel while in 375 µm channels. The results (the reader is referred to Supplementary Video S3) show a focal line forming close to the position of the membrane. This indicates that the membrane has to be considered acoustically transparent (one pressure node at 1 MHz in a 760 µm-wide channel). Although there is a porous membrane in the middle of the 760 µm channel, the results indicate that in the case of the focusing of particles, the permeable membrane acts as a solid wall in the middle, physically dividing the channel into two channels of 375 µm. From the liquid dynamics perspective, the liquid flow and species transport are effectively affected by the presence of the membrane.

From another perspective, the effective width is used to estimate the focal position in a silicon device, with a main channel and an echo channel separated by a thin solid wall, which is defined as the distance that acoustic waves would travel if all regions were considered to contain water. This is calculated as follows [21]:

(1) $$W_{eff}=W_{main}+W_{echo}\left(\frac{C_{w}}{C_{echo}}\right)+W_{wall}(\frac{C_{w}}{C_{si}})$$

where $W_{main}$ is the width of the main channel that is always filled with water, $W_{echo}$ is the width of the echo channel that is only used to affect the focal position of particles in the main channel, $W_{wall}$ is the thickness of the Si barrier, and $C_{w}$, $C_{si}$, and $C_{echo}$ indicate the speed of sound in water, Si, and the fluid, respectively. By dividing the speed of the sound in water by the effective channel width, the resonance frequency, which corresponds to particle focusing, can be calculated. Just as the media of both the main channel and the echo channel are the same, C_echo_ = C_w_, in the same way, C_main_ and C_echo_ have the same width. It is possible to evaluate the effective width of the device, which turned out to be about 752 μm. Such a small difference means that the wall can be considered near-transparent to the propagation of acoustic waves.

Based on past research, a thin (10–20 µm) continuous silicon wall (with no pores) between two channels divides them physically but is also acoustically transparent. It has been observed in the literature that an increase in the thickness of the wall results in an unpredictable focusing position [22].

The movement of particles or cells suspended in a liquid under the influence of acoustic forces within a microfluidic device is controlled by the balance between the drag force and the acoustic radiation force in the lateral direction. The interaction between an acoustic wave and a particle gives rise to the acoustic radiation force, resulting in a net force acting on the particle. The acoustic radiation force scales with the particle volume, enabling size separation of particles. Larger particles experience a higher acoustic radiation force compared to smaller particles, assuming all other factors remain constant [23]. The radiation force for spherical particles is as follows:

(2) $$F_{rad}={4\pi \Phi a}^{3}k_{0}E_{ac}\sin(2k_{0}y)$$

with (a) being the radius of the particle, ($k_{0}$) the angular phase number, (y) the channel width, ($E_{ac}$) the acoustic energy, and Φ the acoustic contrast factor.

As an acoustic field is applied to a liquid, it transfers energy to the liquid through viscous attenuation, leading to the generation of a consistent fluid motion. Variables such as geometry and velocity significantly influence the mechanism of viscous attenuation involved. The streaming in such a device is boundary-driven, meaning that strong vortices along the top and bottom wall of the channel are generated in a region defined as viscous boundary layer thickness. Such vortices then generate the bulk vortices responsible for the streaming effect [24]. Consequently, nonlinear interactions within the boundary layers typically result in a steady rotational flow, manifesting as two vortices at each channel wall in the direction of wave propagation [25].

**Figure 3:**
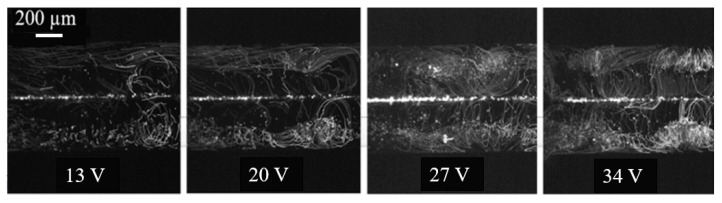
Z-stacking of 1 µm polystyrene particle movement along the vortex flow.

Figure 3 is a visualization of introducing static 1 µm polystyrene particles in both channels separated by membranes and applying acoustic force. The particle trajectories conceal the vortices formed by acoustic streaming. The vortices are more distinct when a higher applied amplitude is used. Comparing this visualization with Figure 2 confirms the effect of the size of the particles on the representation of their migration behavior induced by acoustics. When the 5 µm polystyrene particles are used, the particles are affected by the radiation force. Each channel focuses separately towards the pressure node, while 1 µm polystyrene particles are more influenced by liquid vortices (acoustic streaming) and follow their rotational flow. From Figure 3, it can be noted that the streaming is more pronounced near the outer walls. As stated before, as the wall provides a physical obstacle, the vortices are affected by its presence and its nature (porous versus non-porous).

**Figure 4:**
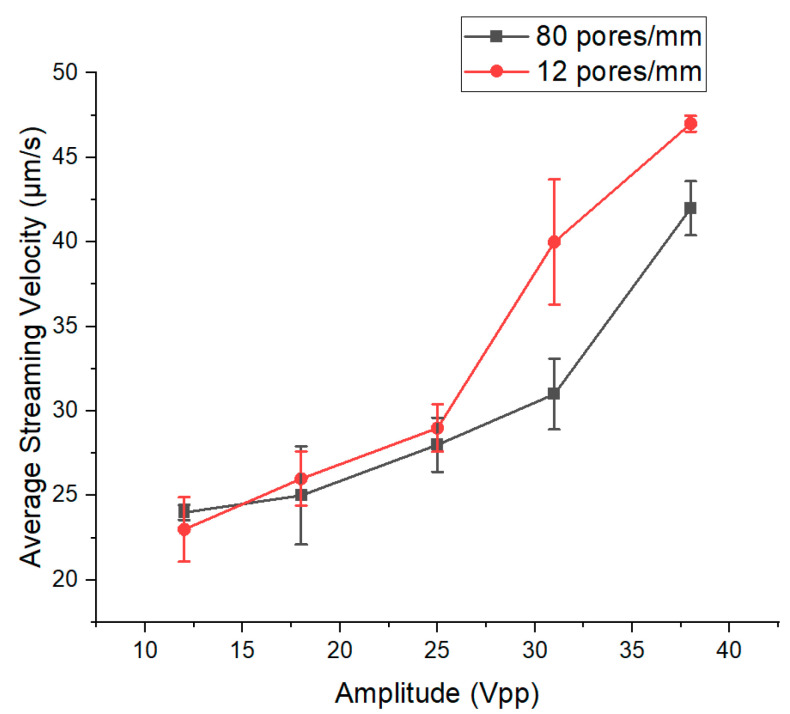
The average magnitude of velocity of flow profiles formed by acoustic streaming in channels with different pore sizes.

The results depicted in these two figures are in complete alignment with the findings reported on the mixing and migration behavior of microparticles in acoustofluidics [26]. Furthermore, the effect of applied potential on the velocity of the vortex flows induced by acoustic streaming was tested in conditions where the membrane had a smaller number of pores and a higher number of pores in the same area analyzed. The pore size stayed the same at 2.5 µm in both situations, and the number of pores was adapted by changing the length of the fabricated pillars that create the porous membrane between the channels. As seen in Figure 4, the number of pores does not play a role in the flow velocity seen in each channel of the microfluidic chip.

In both conditions, the average velocity magnitude of flow vortices increases by increasing the applied potential. This increase from 13 to 27 V is rather subtle and becomes more evident past 27 V. The increase in average flow velocity by increasing the power has already been observed in surface acoustic wave separation systems [27]. Based on past studies, although a positive trend is observed, the increase does not exhibit a linear relationship between the average velocity and the power [28]. Based on past studies, a positive trend of the streaming velocity is observed in line with the applied power. Such an increase does not exhibit a linear relationship between the average velocity and the power. Indeed, as the streaming velocity is connected to the acoustic energy density, such a trend is reported in the literature as a quadratic trend of the streaming velocity in line with the applied power [29]. Instead, the reported data show the streaming velocity has a piecewise linear trend.

**Figure 5:**
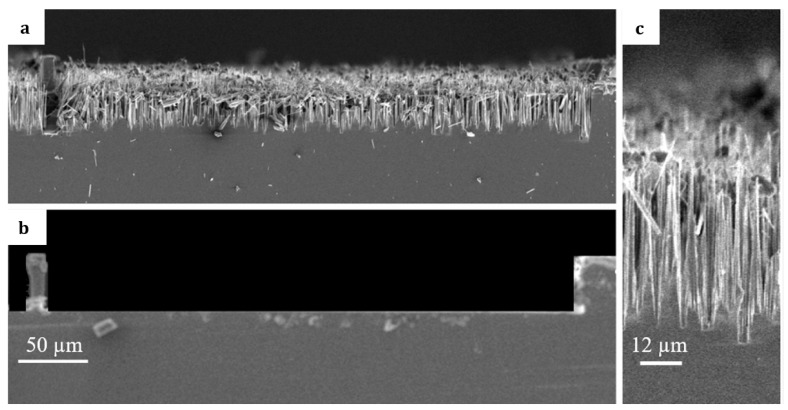
Image taken of one channel of the microfluidic design with the porous wall as a single standing pillar on one side and a solid wall on the other: (**a**) channel bottom covered with so-called black silicon, (**b**) smooth channel bottom without black silicon, and (**c**) zoomed image of black silicon structures formed along the channel.

The effect of the roughness of the silicon channel on the average velocity of the flow-induced acoustic streaming was characterized. The roughness of the channel was in the form of an array of black silicon covering the microfluidic channel. These nanostructures form as a result of the Reactive Ion Etching (RIE) of silicon. In RIE, Fluorine compounds are widely used to modify the surface of silicon wafers [30], and the formation of black silicon occurs due to a local variation of the silicon etch rate. This variation in the etching rate can be caused by multiple factors: the silicon surface itself, such as an inhomogeneous deposited oxide layer, or incompletely or poorly removed native oxide. In addition, the plasma tool and plasma source can also cause a micromasking material by incomplete removal of the etching mask, which can lead to the formation of nanopillars [30,31]. Figure 5 shows SEM images of one channel of microfluidic channels separated by a porous wall. As depicted in Figure 5a,b, the only difference between the two tested microfluidic chips is the existence of a black silicon array in one (two channels covered by black silicon with a membrane in the middle) compared to the other (two channels without the black silicon with a membrane in the middle).

**Figure 6:**
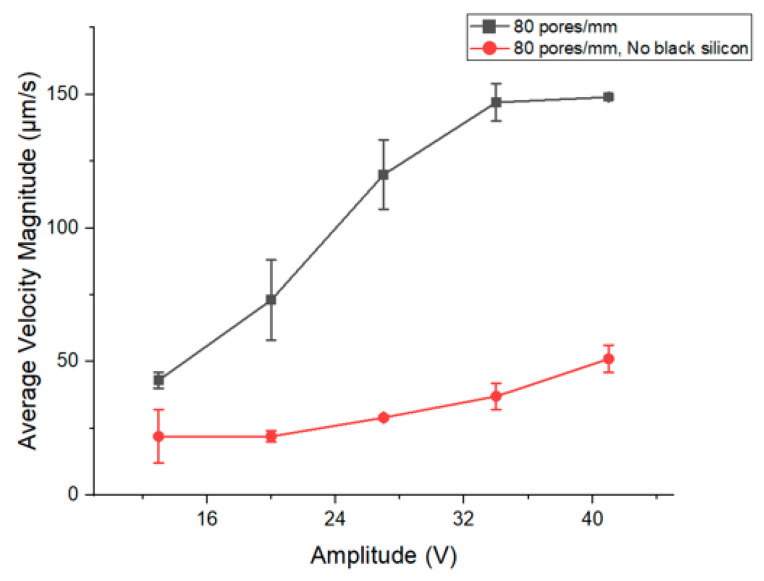
Comparison of average velocity magnitude in the existence of arrays of black silicon inside the channel.

The average velocity of acoustic streaming in the microfluidic chip covered with black silicon is threefold more than a channel with a smooth surface and no black silicon at 41 V. Figure 6 shows the trend in detail. The difference in the flow velocity begins with a subtle inclination, eventually escalating to a notable upsurge.

Although more research is warranted to deepen our understanding of the reason behind this increase in the flow velocity, indeed, the presence of nano-sized black silicon affects the efficiency of acoustic streaming in microfluidics directly and can alter the behavior of particles and fluids in microchannels due to its unique surface properties [32].

From a different view, each black silicon nanostructure can act like a sharply cornered structure along the channel. Past research on the topic shows that rapid and homogeneous mixing inside a microfluidic channel can be achieved via the acoustic streaming phenomenon induced by oscillating sidewall sharp-edged corners. By optimizing the design of the sharp edges, excellent mixing performance can be achieved in the acoustofluidic device [33]. The recirculating flows induced by the oscillation of sharp edges allow fluids to interchange and thus enhance the mass transfer, significantly improving the mixing efficiency. Certainly, the scale of the improvement and increase in the mixing behavior depend highly on the sharp edge geometry and the driving frequency [34]. Experimental measurements performed for tip angles between 10 ° and 90° show that the magnitude of the acoustic streaming velocity decreases with the increase in the tip angle [35].

Acoustic streaming is essential for small particles following vortex flows. If the particles are relatively large, the particle motion is governed by radiation forces. Radiation forces make particles accumulate in fixed points, whereas acoustic streaming makes particles rotate in steady vortices. Large copolymer particles focus in a line followed by the 70° sharp edge tip [36].

Growing silicon grass (black silicon or silicon nanostructures) in a microchannel is difficult. Still, it is achievable using techniques such as RIE with highly controlled etching variables [37] and Metal Assisted Chemical Etching (MACE) [38]. Integrating silicon grass formation into microfluidic channels adds complexity to the fabrication process, as the confined space of a microchannel can make it more difficult to achieve uniform etching and structure formation, which can affect the reproducibility of the microdevice and the silicon grass uniformity of the channels. It should also be noted that despite increasing and improving the channel mixing velocity, these structures significantly alter the fluid dynamics inside the microchip. Therefore, careful considerations need to be made for their use, including the impact on pressure drop and potential fouling within the porous structures.

**Figure 7:**
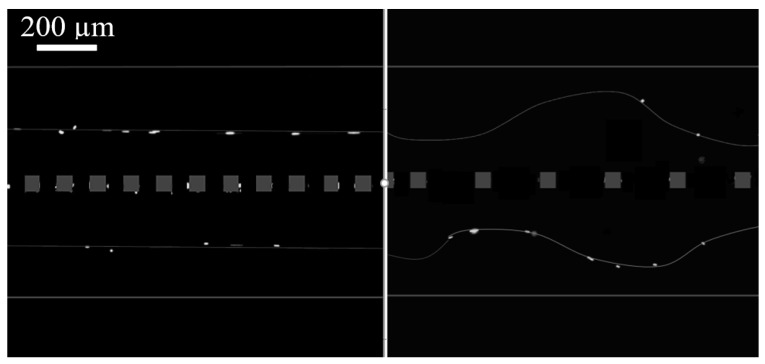
Focusing of 5 µm polystyrene particles when the spacing of pillars in the fabricated membrane is 2.5 µm and membrane thickness is 10 µm (Supplementary Video S3), compared to when the spacing is 5 µm and membrane thickness is 5 µm (Supplementary Video S4). (The grey squares are intended solely to give a better understanding of the membrane position, do not contain factual information, and are not to scale.)

The final characterization of the fabricated acoustofluidic device with a permeable wall in between was to test the effect of pore spacing on the generated acoustic forces. Two chips were used, one with a pore size of 2.5 µm and the other with 5 µm spacing between the silicon pillars that compile the wall between the two channels of 375 µm wide. In each channel, polystyrene particles with the same flow rate were introduced. As Figure 7 exhibits, the spacing on the pores has an effect on the focusing behavior of particles under the influence of the radiation force, hence changing the balance between acoustic radiation force and drag force. When the pore sizes are two times bigger (5 µm), the particle focusing pattern shows a curved behavior instead of focusing in a straight line with 2.5 µm pores.

These results indicate that when the size of the pores in the walls increases to a certain point, the permeable wall no longer acts as before between the two channels, and the pressure difference changes. A pore interferes with the formation of the bulk acoustic standing wave in the channels. The standing acoustic wave field forms a distribution of minimum and maximum pressure regions known as pressure nodes and antinodes. These pressure variations play a crucial role in manipulating particles or cells within the microfluidic channel. Positive and negative acoustic contrast factors determine the direction of the force exerted on particles towards pressure nodes or antinodes, influencing their migration time and behavior [39]. Additional experiments are required to understand the intricacies of the observed phenomena comprehensively.

**Figure 8:**
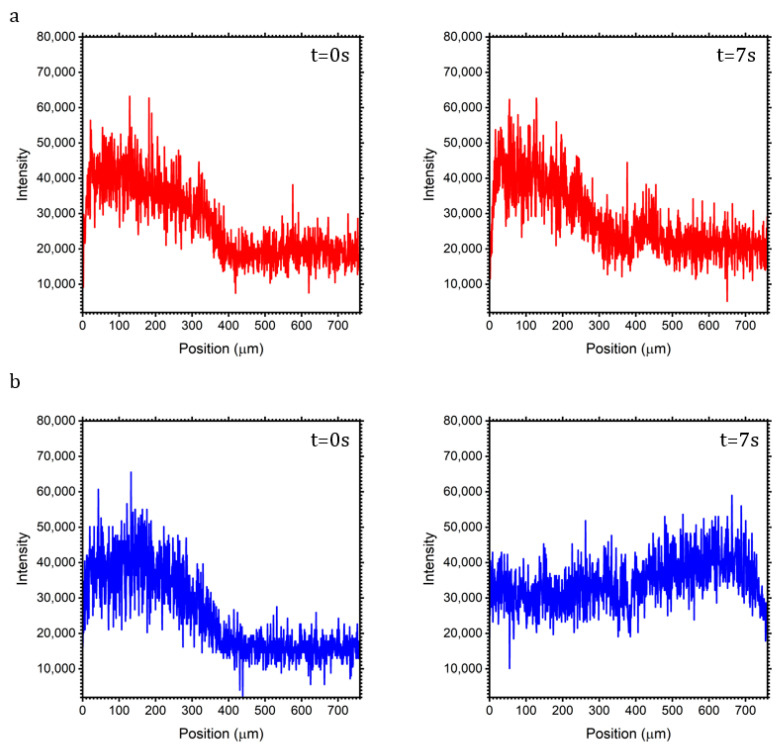
Comparison of fluorescence intensity over the width. (**a**) 1 MHz (Supplementary Video S6); (**b**) 2 Mhz (Supplementary Video S7).

The results of the mixing ability of standing BAWs are presented in Figure 8. A preliminary test without acoustics (see Supplementary Video S5) showed a limited diffusion between the channels. In the case of 1 MHz, the acoustic operation generates microstreaming near the pores. It is clear from the video (Supplementary Video S6) that streaming is confined to a region near the wall. This is also confirmed by the analysis reported in Figure 8a, which shows a peak near the wall. On the other hand, the case of 2 MHz appears to be more efficient compared to the case of 1 MHz. Figure 8b presents the fluorescence intensity in the case of 2 MHz, where the higher degree of mixing is clear, since at time 0 s and time 7 s, the intensity in both channels has changed along almost the entire length. It can be noted, however, that the mixing along the longitudinal direction is not homogeneous. Such an effect could be compensated for by a higher actuation potential, resulting in stronger streaming and concomitant mixing. The reasons behind a more efficient mixing in 2 MHz are related to the number of vortices and symmetry. In the case of 1 MHz, conventional acoustic streaming at fundamental harmonics generated four vortices, while in the case of 2 MHz, which results in the first harmonic, the number of vortices was eight. A higher number of vortices appears to improve mixing. As for the symmetry, even if the wall is transparent to waves, it is not in terms of fluid dynamics. The fact that the wall breaks the streamline may result in an alteration of the vortices’ shape near the wall, leading to worse mixing. Further tests need to be performed to achieve full mixing at a higher potential. In such a case, a temperature controller would be needed, as the temperature increase would no longer be negligible [40].

## 4. Conclusions

An approach was pursued of combining a fabricated membrane in silicon with acoustic forces as an external force. Our findings present that the membrane with 2.5 um pores separates two channels physically but not acoustically and that a resonance frequency can be applied to the device, forming an acoustic standing wave. Based on the characterization made by this study, the two channels connected with porous walls under the acoustic streaming influence act as one 760 μm-wide channel rather than two 375 μm-wide channels in the context of matching the standing pressure wave criteria of the piezoelectric transducer. Therefore, although the pores enhance the streaming velocity, they do not affect the formation of standing pressure waves in the device for the studied designs. However, increasing the size of the pore by a factor of two will result in a different outcome.

The effect of the number of pores per tested length revealed that they do not affect the acoustic mixing behavior of the device. Lastly, introducing an array of black silicon to the bottom of the channel increased the acoustic streaming velocity. The roughness of the wall plays a significant role in mixing compared to smooth channels. A roughened (black silicon) wall results in a threefold increase in average streaming flow compared to a non-porous wall operated in BAW mode.

Acoustically induced flow patterns in this microdevice demonstrated a controlled movement of liquids and particles within each side of the fabricated membrane. To optimize the microdevice further for microfiltration applications, extra adaptations need to be made to the device, such as utilizing a pressure gradient between the two sides of the membrane and surface treatment to avoid fouling.

This investigation provides background information for the flow and transport of particles and fluids in porous media and the high-speed contactless manipulation of micron-sized particles and cells. In addition, the frequency influences the mixing efficiency, defining a better transport in the case of 2 MHz. Such devices find applications where diffusive limited processes are a limiting stage for separation. The small pore sizes of these Microfilter membranes (down to 2.5 for the present study, can be reduced down about 100 nm) allow the effective removal of particles, macromolecules, bacteria, and microorganisms while passing smaller molecules. Characterizing the acoustic-induced flow patterns in this study offers precise control over liquid flow, which allows the optimization of membrane performance and efficiency of the filtration process, and the handling of delicate or complex samples without significant damage.

## Data Availability

The data presented in this study are available on request from the corresponding author. The data are not publicly available due to data volarization trajectory requirements.

## Supplementary Materials

The supplementary videos can be downloaded at https://zenodo.org/records/11353359 (accessed on 28 May 2024).
